# 3D positioning and optically detected magnetic resonance of intracellular fluorescent nanodiamonds using a multi-plane microscope

**DOI:** 10.52601/bpr.2025.250012

**Published:** 2026-02-28

**Authors:** Bowen Zhang, Yichen Yang, Jia Su, Linyu Zeng, Zenghao Kong, Zhijie Li, Zhiping Yang, Fazhan Shi

**Affiliations:** 1 CAS Key Laboratory of Microscale Magnetic Resonance and School of Physical Sciences, University of Science and Technology of China, Hefei 230026, China; 2 Anhui Province Key Laboratory of Scientific Instrument Development and Application, University of Science and Technology of China, Hefei 230026, China; 3 Hefei National Laboratory, University of Science and Technology of China, Hefei 230088, China; 4 School of Biomedical Engineering and Suzhou Institute for Advanced Research, University of Science and Technology of China, Suzhou 215123, China

**Keywords:** Multi-plane microscope, Fluorescent nanodiamonds, Optically detected magnetic resonance, Nitrogen-vacancy center, Single-particle tracking, Correlated quantum sensing

## Abstract

Wide-field quantum sensing with fluorescent nanodiamonds (FNDs) in biological systems offers significant potential for understanding intracellular dynamics at the nanoscale. However, current wide-field quantum sensing methods are limited to 2D correlated measurements. 3D correlated quantum sensing remains challenging due to the inherent properties of wide-field microscopy. Here, we have developed a multi-plane wide-field microscope platform that achieves an imaging volume of 50 × 50 × 5 μm³. This is accomplished by simultaneously imaging eight focal planes at varying sample depths using a beam-splitting prism. By employing a Fourier-transform-based fluorescent particle positioning method, the platform attains lateral positioning precision of 9 nm and axial precision of 12 nm. Using this platform, we performed correlated 3D positioning of FNDs in mouse cardiomyocytes and conducted optically detected magnetic resonance on nitrogen-vacancy color centers within intracellular FNDs. Our results demonstrate the potential of this platform for single-particle tracking and highlight its capability to achieve correlated 3D quantum sensing.

## INTRODUCTION

The dynamics of intracellular fluorescent particles or macromolecules is an important research field in biophysics revealing the heterogeneity of the microenvironment around single particles. During the past decade, a series of imaging techniques have been applied in life sciences: fluorescence recovery after photobleaching (Carnell *et al.* 2015; Saito *et al.* 2023; Tingey *et al.* 2021), fluorescence correlation spectroscopy (Enderlein 2024; Kleusch *et al.* 2020; Sarkar *et al.* 2023), single-molecule displacement mapping (Choi *et al.* 2023; Xiang *et al.* 2020, 2023), single-particle tracking (SPT) (de Messieres *et al.* 2016; Huseyin and Klose 2021; Travers *et al.* 2020), *etc*. While other techniques can only provide statistical information about particle motion (Dominguez-Medina *et al.* 2016; Elson 2011; Haustein and Schwille 2007; Lippincott-Schwartz *et al.* 2001; Sprague *et al.* 2004), SPT can capture the trajectory of individual particles. SPT is capable of investigating the viscous coefficient of the cytoplasm (Madsen *et al.* 2021), the endocytic mechanism of the cell (Ruthardt *et al.* 2011), the targeted binding of drug particles (Watanabe *et al.* 2024) and other biophysical research issues.

**Figure 1:**
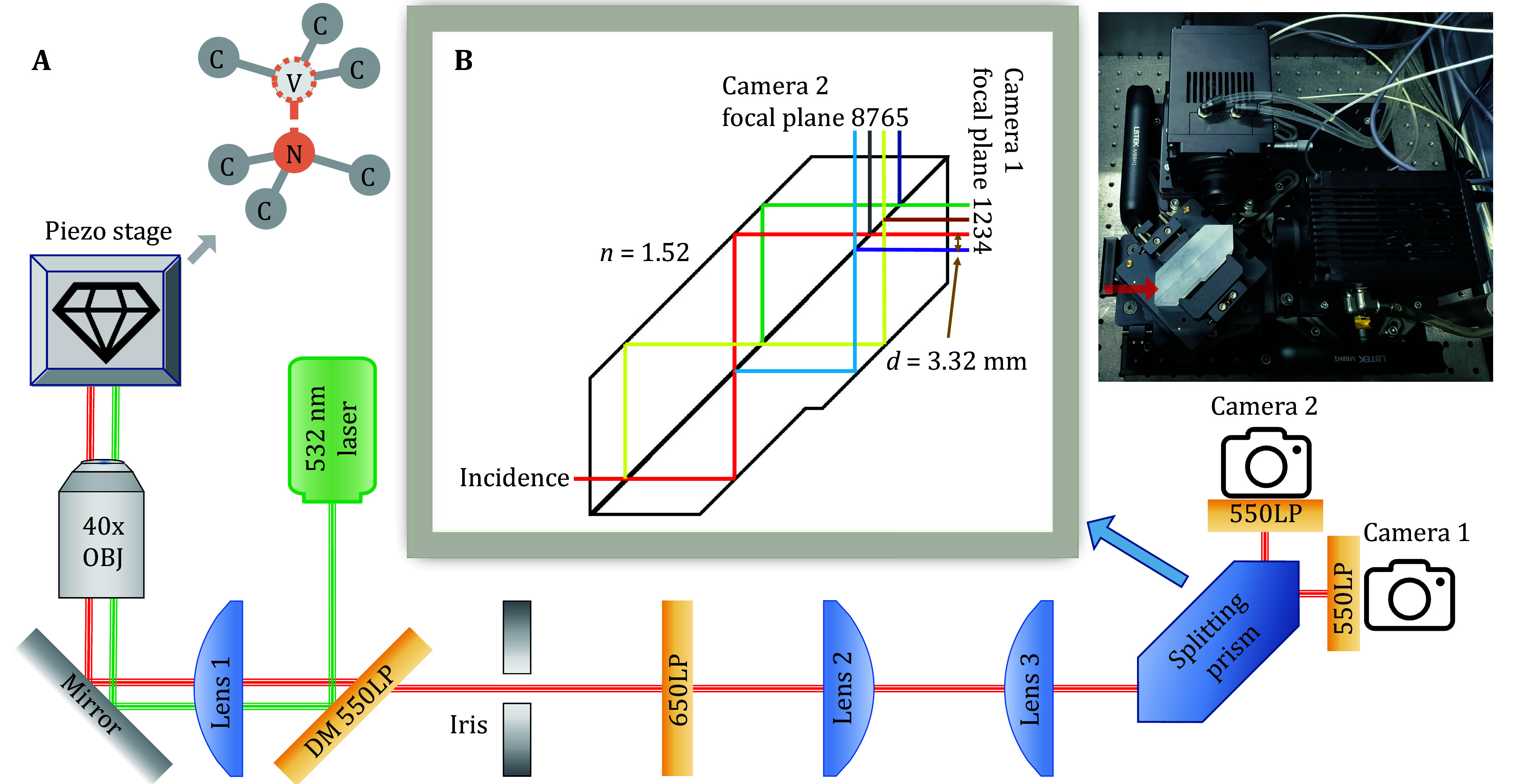
**A** Schematic diagram of multi-plane microscope. The model of the laser is Coherent Verdi G5. OBJ is the microscopic objective (Olympus LUCPLFLN40X). All three lenses (LBTEK MCX10618-B) have a focal length of 250 mm. The iris is an adjustable rectangular slit (LBTEK ASR12) that serves as a field diaphragm. DM is the dichroic mirror (LBTEK DM20-550LP). LP refers to long-pass, and the number before LP is the cut-off wavelength in nanometers. The model of the two cameras is Tucsen Dhyana 400BSI. Next to the piezo stage is a structure diagram of the NV center. **B** Schematic diagram of the beam-splitting prism. The incident beam is divided three times into eight beams and then enters the two cameras. A real picture of the prism part is shown aside, and the red arrow indicates the direction of the incident beam

Early SPT target objects were not fluorescent particles, but scattering particles, such as polystyrene pellets and colloidal gold particles (Sheetz *et al.* 1989). Scattering particles have limitations in biological systems due to their large size and the anisotropy of Rayleigh scattering (Manzo and Garcia-Parajo 2015). As more and more fluorescent materials were invented (Zheng *et al.* 2023), the objects of SPT were gradually shifting to fluorescent particles (Liebel *et al.* 2020). However, many organic fluorescent materials face photobleaching and photoblinking under laser irradiations (Hu *et al.* 2022), which contradicts the advantages of SPT to track individual particles for long-term dynamic studies. In 2008, Chang *et al*. achieved mass production of FNDs and used them for SPT within HeLa cells (Chang *et al.* 2008). FNDs are ideal for intracellular SPT, with high fluorescence intensities, long-term photostability and favorable biocompatibility (Doherty *et al.* 2013). Fluorescence of FNDs is contributed by the nitrogen-vacancy (NV) color centers (Nizovtsev *et al.* 2001), a type of point defect in diamond: one carbon atom is replaced by a nitrogen atom, and one of four adjacent carbon atoms is vacant (Fig. 1A). The electrons of the NV center constitute a spin 1. The electron spin of NV centers in FND can be optically initialized and read out with coherent manipulation by microwave under ambient conditions (Jelezko *et al.* 2004). This technique of detecting the quantum states of spin in solids via the optical method is known as optically detected magnetic resonance (ODMR). The Hamiltonian of the NV center contains physical quantities such as electric field, magnetic field, temperature, stress, spin coupling, *etc*., and the physical information of the surrounding environment can be obtained during the evolution of the quantum state (Loretz *et al.* 2013). The NV center is not only a fluorescent label, but also a nanoscale quantum sensing probe, which is widely used in single-molecule magnetic resonance (Du *et al.* 2024; Shi *et al.* 2015), free radical detection (Nie *et al.* 2021), intracellular thermometry (Kucsko *et al.* 2013) and other biophysical applications.

The SPT of FNDs is mainly demonstrated by single-pixel detectors such as single photon avalanche photodiode, however, it is challenging to study the dynamics of multiple sensors simultaneously to further reveal the correlation between the labels. With the development of CCD and sCMOS, more and more SPT works adopted wide-field microscopes for better acquisition frame rate and imaging range than confocal microscopes. As an example, Simon Haziza *et al*. used a CCD camera to track FNDs within mouse neurons with a frame rate of 20 FPS and an imaging range of 50 × 50 μm², where the tracking range was limited to the 2D plane (Haziza *et al.* 2017). If obtaining the 3D imaging range by repetitive focusing, the frame rate will be greatly reduced. Then, Descloux *et al*. designed a multi-plane wide-field microscope to achieve 3D phase retrieval and super-resolution optical fluctuation imaging (Descloux *et al.* 2018). The microscope used a special beam-splitting prism to simultaneously image eight focal planes at different depths without any mechanical movement. A couple of years later, Louis *et al*. used a similar microscope to track polystyrene fluorescent pellets in water to study the hydrodynamics of the pellets captured by an optical trap (Louis *et al.* 2020, 2023). The most important advantage of this multi-plane platform over traditional wide-field microscope is high temporal resolution in 3D imaging. The platform can image a 3D space at the camera's maximum frame rate (up to 200 FPS), while traditional ones suffer a significant frame rate drop due to repetitive focusing. The adoption of the multi-plane microscopy and ODMR will offer a new insight: intracellular quantum sensing in 3D to investigate not only the dynamics but also the correlation of the labels in the cells. In this work, we chose FNDs as the target particles. Compared with polystyrene fluorescent pellets, the inhomogeneity of FNDs (Reineck *et al.* 2019) posed a challenge to data processing. Using a modified Fourier-transform-based positioning algorithm (Martens *et al.* 2018), we achieved 3D positioning of intracellular FNDs. We also performed ODMR experiments on intracellular FNDs, and demonstrated the potential of the platform to track multiple particles in parallel. The demonstrations offer a new possibility to perform intracellular temperature and magnetic field sensing while tracking single particles.

## MULTI-PLANE WIDE-FIELD MICROSCOPE PLATFORM

### Platform setup

The multi-plane platform is based on the traditional wide-field microscope (Fig. 1A), with the addition of a specially designed beam-splitting prism (Fig. 1B). The beam-splitting prism splits eight focal planes of different depths and projects the separated images onto the CCD simultaneously (Descloux *et al.* 2018) with an axial imaging range of about 5 μm. Each part of the experimental platform will be described in detail in the following paragraphs.

A continuous wave laser generates an approximate Gaussian beam with a wavelength of 532 nm. The laser beam is reflected on the 550 nm long-pass dichroic mirror, and focused by the wide-field lens. The laser beam is focused accurately to the back focal point of the objective lens, and then, emits parallelly from the objective, introducing a wide-field illumination. The wide-field lens and the objective lens form conjugation at infinity, and the illumination spot size can be calculated by:

$ d=D\times\frac{f_{obj}}{f_{lens}}=72\; {\mathrm{\mum}} \;,$

where *d* is the illumination spot diameter, *D* = 4 mm is the incident beam diameter, $ {f}_{obj} $ = 180 mm / 40 = 4.5 mm and $ {f}_{lens} $ = 250 mm are focal lengths of the objective and wide-field lens. The objective lens has a nominal magnification of 40X, and the corresponding tube lens focal length is 180 mm. The illumination spot size matches the imaging field of view, illuminating the entire field of view.

The sample fluorescence is collected by the same objective lens, and then transmitted through three lenses to the sCMOS. The theoretical lateral and axial magnification is given by:

\begin{equation*}\begin{split} &
\beta =\frac{{f}_{1}\times {f}_{3}}{{f}_{obj}\times {f}_{2}}=55.56\;,\\&
\alpha ={\beta }^{2}=3086\;,
\end{split}\end{equation*}

where * β * and * α * are lateral and axial magnification, $ {f}_{obj} $ = 4.5 mm is the objective focal lengths, and $ {f}_{\mathrm{1,2},3} $ = 250 mm are the focal lengths of the three lenses from left to right in Fig. 1A. Note that the leftmost lens is also the wide-field lens. The above magnification values are all theoretical values, and the exact magnification values will deviate slightly due to the error in the position of optical components. All three lenses are plano-convex, and the spheres are facing the side of the parallel ray. Spherical aberration is minimized in this setting. All three lenses are also covered with an antireflective coating of 700−1100 nm (LBTEK B-type coating), so that the transmission rate of sample fluorescence is increased.

In order to ensure that images of different focal planes do not overlap on the camera target plane, a field diaphragm needs to be set in the intermediate real image plane. We choose an adjustable rectangular slit as the field diaphragm, thus precisely limiting the field of view. The slit is mounted on a one-dimensional linear stage, and can translate along the optical axis. Since there are eight intermediate focal planes with different axial positions, the field diaphragm cannot coincide with all of them. The slit is set in the middle of the intermediate planes, so that vignetting around the focal planes is minimized.

Multiple focal planes are introduced via the beam-splitting prism (Fig. 1B). There is a beam-splitting plane in the middle of the prism with a beam-splitting ratio of 50:50. The incident beam is split and then totally reflected on the outer surface of the prism. The reflected rays are split again on the beam-splitting plane. Eventually, an incident beam is split into eight that exit the prism from two perpendicular directions and enter the two cameras. Two adjacent focal planes of the same camera are horizontally spaced 3.32 mm apart. The interval between the two adjacent focal planes in the object space is given by:

$ \delta=\frac{d}{n\alpha}=0.71\; \mathrm{\mum}\;, $

where * δ * is the interval, *d* = 3.32 mm is the horizontal spacing of outgoing beams, *n* = 1.52 is the refractive index (measured at 587 nm), * α * = 3086 is the axial magnification. The above calculation is only a theoretical prediction, and experimental measurements of the exact values are given in the next section. Two cameras are mounted on two linear stages, so that it is possible to adjust the relative axial position of the two sets of four focal planes. The cameras use 550 nm long-pass filters as windows to shield the scattered laser in the environment. In order to reduce the imaging background, a 650 nm long-pass filter is also set in the optical path.

The sample stage is composed of coarse and fine adjusting parts. The coarse adjusting part is an XY linear translation stage (Newport M-406 XY) with servo motor actuators (Thorlabs Z812B). The linear stage has a moving range of about 10 mm, which is enough for coarse adjustment of sample position. There is a through hole in the middle of the translation stage to allow the objective lens to pass through. The fine adjusting part is an XYZ piezo scanner (CoreMorrow P15.XYZ300S-C2) with a single axis range of 300 μm. The piezo scanner communicates with the computer by serial port and can move in a specified sequence controlled by a Python program. An Ω-shaped microwave antenna is fixed to the scanner for ODMR experiments. Microwave signals are generated by a signal generator (Ceyear 1435F), and amplified by an amplifier (Mini-Circuits ZHL-50W-63+). The microwave is switched on and off via a microwave switch (Mini-Circuits ZASWA-2-50DRA+), which receives TTL signals from a high-speed TTL signal generator (SpinCore PBESR-PRO-500-PCI).

### Focal planes calibration

**Figure 2:**
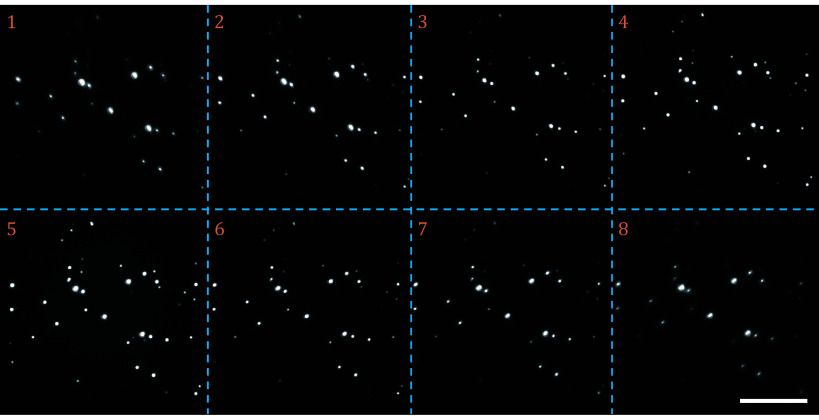
Raw image of FNDs (Adamas ND100) dispersed on a cover slip. The first row is the focal planes 1−4 of camera 1, and the second row is the focal planes 5−8 of camera 2. The *Z* position of the sample is adjusted to the middle of the eight focal planes. The scale bar corresponds to 20 μm

For accurate 3D positioning of fluorescent particles, the parameters of eight focal planes must be calibrated first. FNDs dispersed on a cover slip (Fig. 2) are used for focal plane calibration. We choose a relatively bright FND as the calibration particle, and adjust the sample stage so that the calibration particle is in the middle of the field of view. Then the Python program controls the piezo scanner to move 20 μm along the *Z*-axis with a step length of 0.1 μm, and sends capture triggers to the cameras at each position.

**Figure 3:**
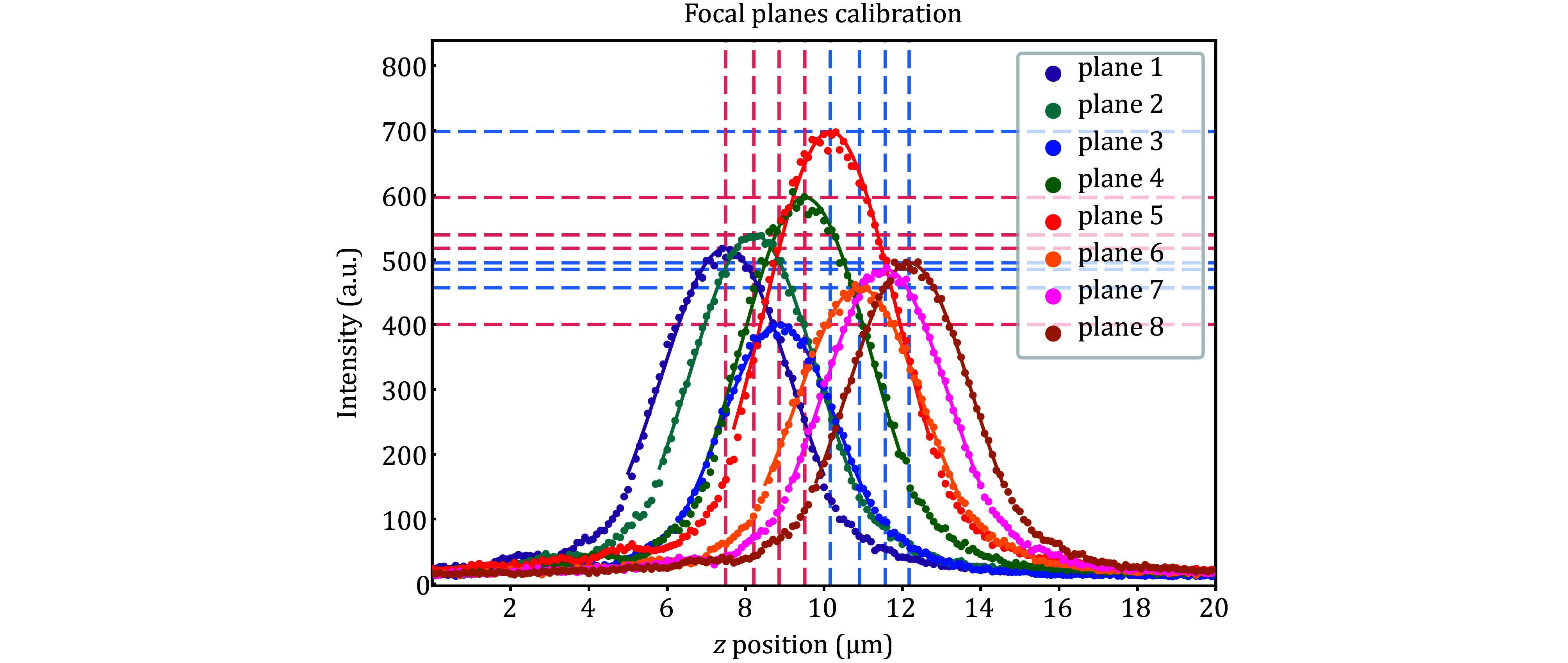
Focal plane calibration. The horizontal axis is the *Z* position of the piezo scanner, and the vertical axis is the fluorescence intensity of the ROI. The eight color data points correspond to eight focal planes, and the curves are the Gaussian fitting of the data points. The vertical dashed lines are the *Z* positions of the focal planes, and the horizontal dashed lines are the relative brightness of the focal planes. Red and blue dashed lines correspond to camera 1 and camera 2. The difference in brightness between focal planes comes from the beam-splitting non-uniformity of the prism. The reflectivity of the beam-splitting prism is slightly larger than the transmissivity, so the focal plane with more reflections than transmissions during splitting is brighter. The focal plane 5 has the highest brightness after three reflections and zero transmissions; The focal planes 1, 2 and 4 have relatively high brightness after two reflections and one transmission; The focal planes 6, 7 and 8 have relatively low brightness after one reflection and two transmissions; The focal plane 3 has the lowest brightness after zero reflection and three transmissions. The slight difference in brightness among focal planes 1, 2, 4 and 6, 7, 8 originates from the wavelength-dependent beam-splitting ratio

From the above image sequence, we first obtain the *Z* position and relative brightness of the focal planes (Fig. 3). We start by extracting the region of interest (ROI) of the calibration particle on all focal planes. The average of the maximum per row of the 2D gradient of the ROI is calculated as the intensity. Compared with the raw value, the gradient is less affected by the background and decays faster when out of focus (Louis *et al.* 2020). A Gaussian fit is performed near the focal position of each focal plane. The peak position corresponds to the focal plane Z position and the peak height corresponds to the relative brightness. In this work, the two cameras are set to extended mode, *i*.*e*. the focal planes of the two cameras are arranged separately. The two cameras can also be set to interlaced mode (supplementary Fig. S1) to improve the *Z*-direction positioning accuracy while reducing the *Z*-direction imaging range.

The *X*-*Y* coordinate systems of different focal planes are not aligned, meaning that pixels with the same *X*-*Y* coordinates in different focal planes correspond to different real *X*-*Y* positions. This deviation comes from factors including the discreteness of the pixels, the difference in the lateral magnification of the focal planes, and the relative rotation between the two cameras. In order to reduce this deviation, an affine calibration between focal planes is required. First, we select an image in the middle of two adjacent focal planes, that is, two focal planes are equally out of focus on this image. Then we recognize all fluorescent particles in both two focal planes based on the threshold and local maximum, and use an improved Munkres algorithm (Munkres 1957) to pair the same particles in the two focal planes. After particle pairing, a constrained 2D affine transformation is added between the two focal planes to minimize the sum of the distances between all particle pairs. A complete 2D affine transformation has six parameters, including two translation parameters, one rotation parameter, two scaling parameters and one shearing parameter. Here, we consider the scaling to be isotropic and that there is no shearing, so the transformation parameters are reduced to four. A constrained affine transform can be written as:

$ \left(\begin{array}{c}{x}_{1}\\ {y}_{1}\end{array}\right)=scale\times \left(\begin{array}{cc}\mathrm{cos}\theta & -\mathrm{sin}\theta \\ \mathrm{sin}\theta & \mathrm{cos}\theta \end{array}\right)\left(\begin{array}{c}{x}_{0}\\ {y}_{0}\end{array}\right)+\left(\begin{array}{c}dx\\ dy\end{array}\right) \;,$

where $ \left({x}_{0},{y}_{0}\right) $ and $ \left({x}_{1},{y}_{1}\right) $ are the coordinates before and after the transformation, * θ * is the rotation angle, * scale * is the scaling factor, * dx * and * dy * are translation parameters. The affine transformation parameters between each of the two adjacent focal planes are calculated and then combined with the intermediate datum focal plane (plane 5 in this work). We obtain the complete focal plane calibration parameters for the 3D positioning of fluorescent particles (supplementary Table S1). The lateral magnification of the focal plane closer to the objective lens is larger and therefore requires a smaller scaling factor to correct. Also, there is a relative rotation of about 0.003 rad between the two cameras.

## 3D positioning method of fluorescent particles

We adopt a fluorescent particle positioning method based on 2D Fourier transform coefficients (Martens *et al.* 2018), which is faster compared with Gaussian fitting. Before positioning, we first recognize all the fluorescent particles in the image stack and cut corresponding ROIs. The image stack is the 3D array of eight focal planes stacked sequentially, and the intensity value of each plane is divided by its relative intensity in supplementary Table S1. This intensity normalization is necessary for *Z*-direction positioning. The ROI side length is set to 15 pixels which can cover the particle without introducing too much background. We acquire the size of an ROI is 15 × 15 × 8 pixels.

For each ROI, a 2D Fourier transform is performed on each plane. The complex coefficients with index (0, 1) and (1, 0) are denoted by phasor *X* and phasor *Y*. The sum of the magnitude of the two phasors is positively correlated with the degree of focusing. A Gaussian fitting is performed on the magnitude sum and the *Z* position of the focal planes, and the fitted peak position is the *Z* position of the particle. *X*-*Y* positioning is performed in the focal plane closest to the particle in the *Z*-direction. The *X*-*Y* position in the ROI is linearly related to the argument of the phasors. Finally, the *X*-*Y* position is aligned to the datum focal plane by an affine transformation.

The precision of particle positioning is defined as the standard deviation of repeated positioning of stationary particles. The exposure time is set to 100 ms, and a total of 1000 images are taken. The statistical results of all 31 FNDs in the imaging range show that the lateral precision is 9 nm and the axial precision is 12 nm. The piezo stage moves with FNDs at equal intervals along an axis through the whole imaging range, and the deviation of the fitting position from the real position is defined as accuracy (supplementary Figs. S2 and S3). The real position is defined as the linear fit of the fitted position and image index, and the slope is fixed to the step length of the piezo stage. Under this condition, the lateral accuracy is 11 nm and the axial accuracy is 33 nm. When the exposure time is reduced to 5 ms, the positioning precision and lateral accuracy become worse, but the axial accuracy almost remains the same (Table 1). More detailed statistical results are in supplementary Figs. S4 and S5.

**Table 1:** Precision and accuracy of particle positioning

	Precision			Accuracy	
	100 ms	5 ms		100 ms	5 ms
Lateral	9 nm	14 nm		11 nm	15 nm
Axial	12 nm	25 nm		33 nm	32 nm
Note: The laser power density on the sample is approximately 2 × 10^7^ W/m^2^; The typical value of photon count is 1 × 10^6^ (exposure 100 ms) and 5 × 10^4^ (exposure 5 ms)					

## EXPERIMENTAL RESULTS

### 3D positioning of intracellular FNDs

**Figure 4:**
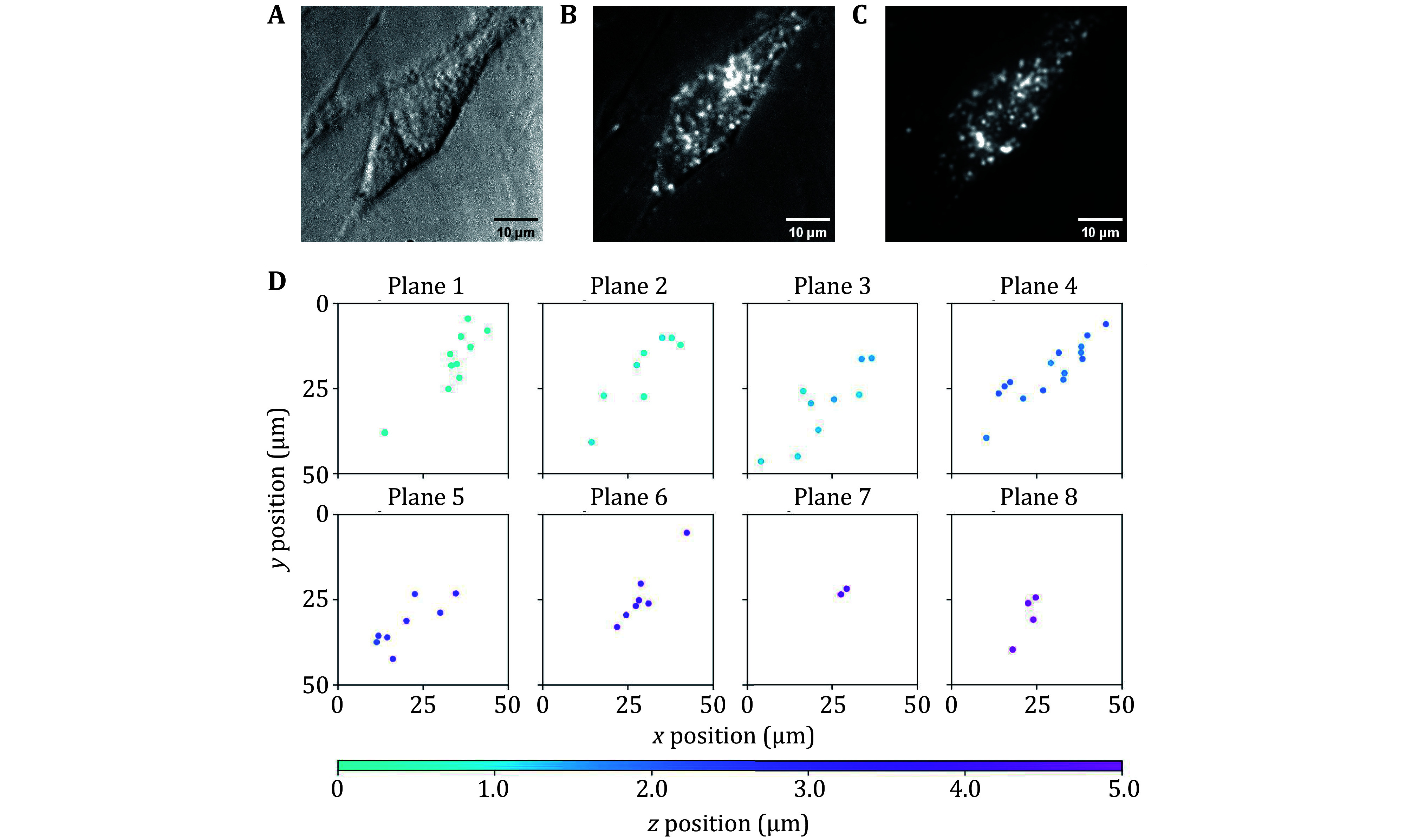
3D positioning of intracellular FNDs in mouse cardiomyocyte (HL-1). **A** Bright-field imaging using an LED light source. **B** Combined bright-field and fluorescence imaging. **C** Fluorescence imaging using a 532 nm laser light source. **D** 3D positioning result. Each FND is displayed on the closest focal plane. The color bar represents the *Z* position

FNDs (Adamas ND100) with an average particle size of 100 nm were fed to mouse cardiomyocytes (HL-1). The particle size was determined by the supplier's dynamic light scattering (DLS) experiments. A Petri dish containing adherent cells was placed under the multi-plane microscope for observation (Figs. 4A−4C), and an LED light source was used for bright-field imaging to find cells. Once an adherent cell containing FNDs was found, all FNDs were positioned by the method in the previous section (Fig. 4D). Each FND was shown on the closest focal plane, while a more precise *Z* position was given by the color bar. The positioning result showed that FNDs were distributed in 3D within the cell. The positioning method of our platform required only two images (one for each of the two cameras), making it more time-efficient compared with traditional confocal or single-plane wide-field microscopes. Our platform did not require any mechanical movement when capturing images, reducing the instability caused by moving components.

### ODMR of intracellular FNDs

**Figure 5:**
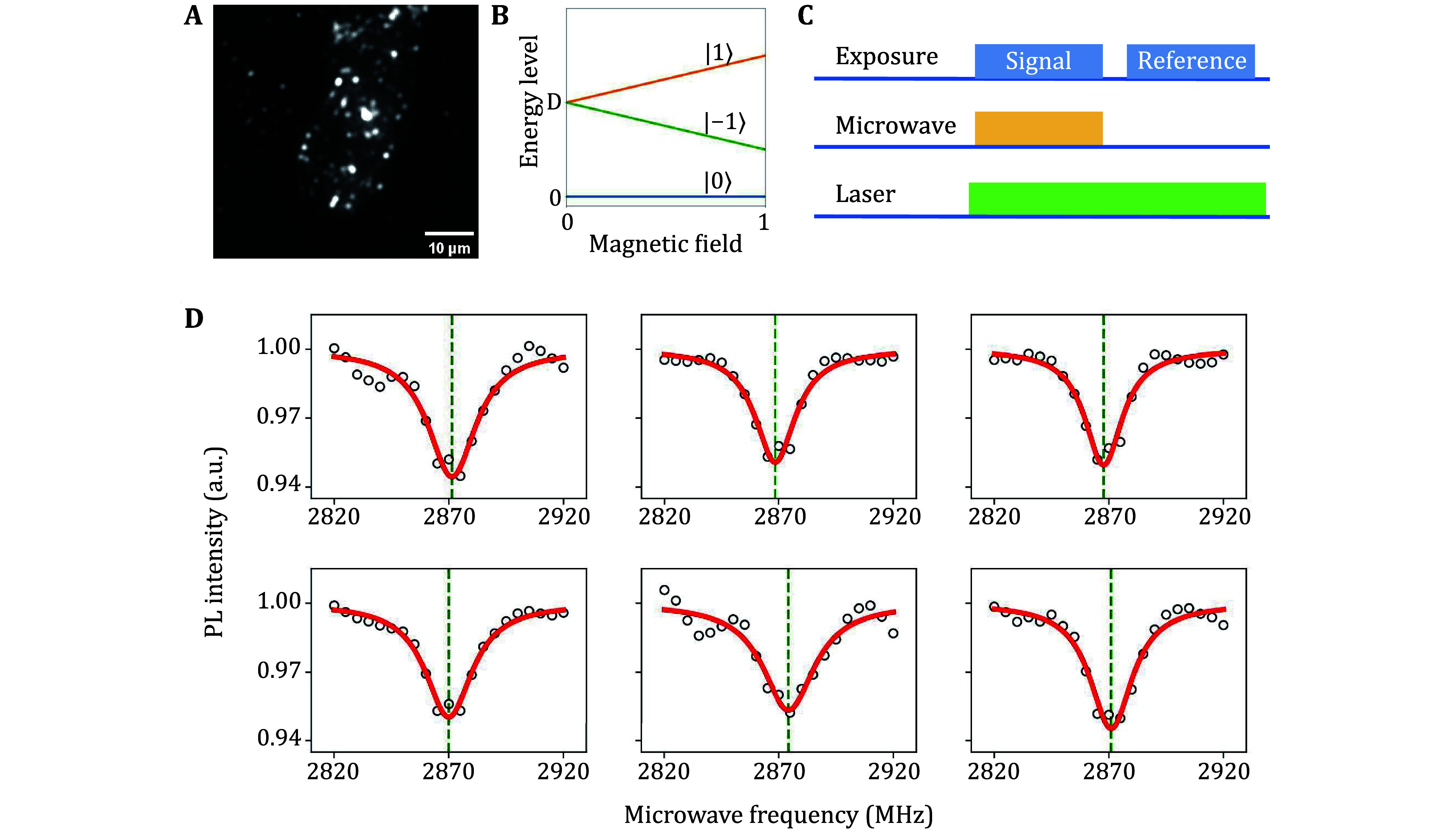
ODMR measurement of intracellular FNDs in mouse cardiomyocytes (HL-1). **A** Fluorescence imaging using a 532 nm laser light source. **B** Energy level of the NV center. *D* = 2870 MHz is the zero-field splitting. When applying a magnetic field along the NV axis, the energy levels of states |−1> and |1> are separated. **C** The measurement sequence of the CW spectrum. The microwave is turned on when capturing the signal, and is turned off when capturing the reference. The laser is always turned on. **D** CW spectrums of intracellular FNDs measured in parallel. The red solid curves are Lorentz fit of the data points, and the green vertical dashed lines are the fitted resonant frequencies

The fluorescence of FNDs originates from the NV center, which is widely used in quantum sensing with ODMR technology. The ground state of the NV center has three energy sublevels, as the spin quantum number of the ground state is one. These three energy sublevels are distinguished by magnetic quantum numbers as |−1>, |0>, |1>. The energy of the |0> is the lowest, and the |−1> and |1> are usually degenerated with an energy difference of about 2870 MHz from |0> (Fig. 5B). This energy difference is usually called zero-field splitting and is in the microwave band, meaning we can manipulate the spins using the microwave. When excited by a 532-nm laser, the fluorescence intensity of |0> is higher than |−1> and |1>. Applying microwave with the frequency near the zero-field splitting can shift the distribution of |0> to |−1> and |1>, resulting in a decrease in fluorescence intensity (Gruber *et al.* 1997). An experiment to measure the zero-field splitting is the continuous wave (CW) spectrum, which is also commonly used for temperature and static magnetic field sensing.

We performed CW spectrum measurement on FNDs inside the HL-1 cell (Fig. 5). Microwave sweeping from 2820 MHz to 2920 MHz was applied to the sample, and the fluorescence intensity was recorded as the CW spectrum. After capturing an image as the signal of each frequency, an image without applying microwave was captured as a reference (Fig. 5C). The normalized fluorescence intensity was defined as the signal divided by the reference. The spectrum was measured at 100 loops in order to reduce the noise. Figure 5D shows the CW spectrum of part of FNDs, and full data are shown in supplementary Fig. S6. All FNDs showed a decrease in fluorescence around 2870 MHz. The fluorescence intensity of some FNDs increased at 2870 MHz due to the energy level splitting of the **|**−1> and **|**1> states caused by the FNDs’ intrinsic stress. This experiment demonstrated the potential of the platform for intracellular temperature sensing.

### Fast tracking of FNDs

**Figure 6:**
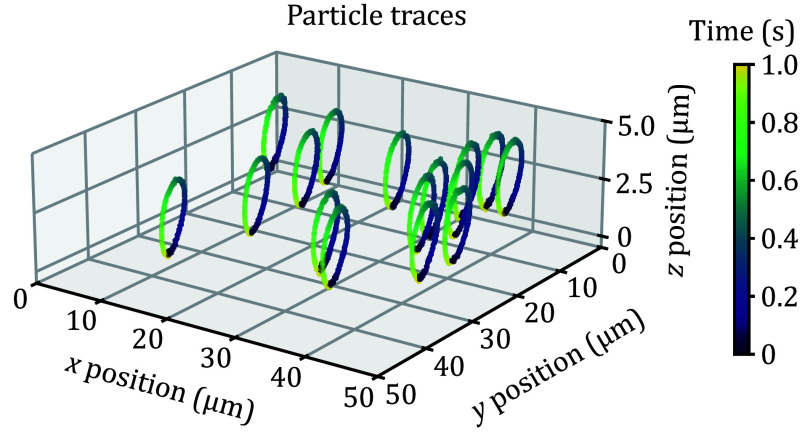
Parallel tracking of FNDs. The piezo stage drives the FND sample to perform a simple harmonic motion in all axes with an amplitude of 3 μm and a frequency of 1 Hz. The capture frame rate is 160 FPS and the exposure time is 5 ms. The color bar represents the time

The multi-plane microscope also demonstrated the ability of SPT. The sample of FNDs dispersed on a cover slip was driven by the piezo stage to do simple harmonic motion. The maximum linear velocity of the movement was about 10 μm**/**s. The cameras captured images at a frame rate of 160 FPS and an exposure time of 5 ms. All FNDs in each image were recognized and positioned, and then matched by the Munkres algorithm (Munkres 1957). An additional check was added to eliminate illegal matches from the Munkres algorithm. Only the FNDs that were tracked from beginning to end were retained, as shown in Fig. 6. Our multi-plane wide-field microscope allowed 3D tracking of multiple particles simultaneously at a high frame rate compared with confocal or single-plane wide-field microscopes. The tracking algorithm was time-consuming and could be significantly accelerated by multi-process parallel computing.

## SUMMARY AND DISCUSSION

In this work, we built a multi-plane wide-field microscope with an imaging range of 50 × 50 × 5 μm³ by simultaneously imaging eight different focal planes. Different imaging ranges can be obtained by adjusting the focal lengths of the objective and lenses in the optical path. Using the Fourier-transform-based fluorescent particle positioning method, the lateral positioning precision achieved 9 nm while the axial precision was 12 nm. This method allows for the simultaneous positioning of all FNDs in the imaging range, and then, enables single particle tracking in 3D. The tracking frame rate was 160 FPS which was limited by the camera readout speed.

ODMR measurements were also performed on the experimental platform. It is possible to extract temperature information from the CW spectrum of intracellular FNDs. It also provides a new insight to perform orientation tracking of individual FNDs in 3D. The correlation measurement from this technique can provide strong support for studying correlated intracellular dynamics of FNDs, such as translational and rotational properties of FNDs on phospholipid membranes.
